# Osteogenesis of Multipotent Progenitor Cells using the Epigallocatechin Gallate-Modified Gelatin Sponge Scaffold in the Rat Congenital Cleft-Jaw Model

**DOI:** 10.3390/ijms19123803

**Published:** 2018-11-29

**Authors:** Satoshi Sasayama, Tomoya Hara, Tomonari Tanaka, Yoshitomo Honda, Shunsuke Baba

**Affiliations:** 1 Department of Oral Implantology, Osaka Dental University, 8-1, Kuzuhahanazonocho, Hirakata, Osaka 573-1121, Japan; sasayama@cc.osaka-dent.ac.jp (S.S.); hara-t@cc.osaka-dent.ac.jp (T.H.); baba-s@cc.osaka-dent.ac.jp (S.B.); 2 Graduate School of Science and Technology, Kyoto Institute of Technology, Matsugasaki, Sakyo-ku, Kyoto 606-8585, Japan; 3 Institute of Dental Research, Osaka Dental University, 8-1, Kuzuhahanazonocho, Hirakata, Osaka 573-1121, Japan

**Keywords:** plant polyphenol, EGCG, gelatin, bone formation, congenital bone defect, dedifferentiated fat cell, adipose-derived stem cell, scaffold

## Abstract

Cost-effective and functionalized scaffolds are in high demand for stem-cell-based regenerative medicine to treat refractory bone defects in craniofacial abnormalities and injuries. One potential strategy is to utilize pharmacological and cost-effective plant polyphenols and biocompatible proteins, such as gelatin. Nevertheless, the use of chemically modified proteins with plant polyphenols in this strategy has not been standardized. Here, we demonstrated that gelatin chemically modified with epigallocatechin gallate (EGCG), the major catechin isolated from green tea, can be a useful material to induce bone regeneration in a rat congenial cleft-jaw model in vivo when used with/without adipose-derived stem cells or dedifferentiated fat cells. Vacuum-heated gelatin sponges modified with EGCG (vhEGCG-GS) induced superior osteogenesis from these two cell types compared with vacuum-heated gelatin sponges (vhGS). The EGCG-modification converted the water wettability of vhGS to a hydrophilic property (contact angle: 110° to 3.8°) and the zeta potential to a negative surface charge; the modification enhanced the cell adhesion property and promoted calcium phosphate precipitation. These results suggest that the EGCG-modification with chemical synthesis can be a useful platform to modify the physicochemical property of gelatin. This alteration is likely to provide a preferable microenvironment for multipotent progenitor cells, inducing superior bone formation in vivo.

## 1. Introduction

Despite the rapid advancement in stem-cell-based therapy, repairing congenital bone defects is still a challenge in dental, maxillofacial, orthopedic, and plastic surgery [1,2,3]. Promising scaffolds that provide a favorable microenvironment for cells are highly desired for treating these diseases in regenerative medicine [4,5]. Various materials have been used as building blocks for scaffolds, such as inorganic materials, synthetic, and natural degradable polymers, including gelatin [5,6,7,8,9]. Numerous strategies have been adopted to functionalize these materials by the alteration of physicochemical and mechanical properties [10], by surface modification [11,12], and by adding additives, such as growth factors and small molecules [9,13]. Functionalization successfully endows the materials with an active interface, which enhances cell migration and proliferation, enabling the differentiation of the progenitor and stem cells [14]. Furthermore, functionalization minimizes host tissue inflammation in the microenvironment [9]. However, the high cost of the procedure is a limiting factor for further functionalization of the scaffold [15,16].

Plant polyphenols are ubiquitously present in our daily diet, food, and beverages. These molecules are relatively cost-effective, when obtained in an economically feasible way [17,18]. Among these polyphenols, epigallocatechin gallate (EGCG), the major polyphenol in green tea, has received significant attention as a prospective health agent due to its diverse pharmacological effects, such as antioxidant [19], anti-inflammation [19], anti-bacterial [20], anti-viral [21], as well as safety [22] and cost effectiveness [18]. It is widely accepted that this molecule exhibits protective properties against cancer [23] and many chronic diseases, such as cardiovascular disease, obesity, and neurodegenerative diseases [24,25,26]. Nevertheless, until 2010, few studies had successfully demonstrated the bone regeneration ability of EGCG with respect to the applicability of its local delivery in bone regenerative therapy, despite its potent ability to induce osteoblastic differentiation in vitro [27]. To address this issue, several researchers, including our group, have addressed the application of EGCG for bone regeneration without the use of other drugs [28,29,30,31]. While other groups have utilized unmodified EGCG mixed with polymers or calcium phosphates, we have reported that gelatin chemically modified with EGCG (EGCG-modified gelatin sponge: EGCG-GS) showed superior bone regeneration ability compared to a gelatin sponge without EGCG and with non-chemically modified EGCG in a critical-sized defect model of mouse calvaria [29]. More recently, we have demonstrated that vacuum-heated EGCG-GS (vhEGCG-GS) induced greater bone formation than did EGCG-GS and a vacuum-heated gelatin sponge (vhGS) in a critical-sized defect model of rat calvaria [32]. Furthermore, the bone-forming ability of vhEGCG-GS is superior to that in the implantation of autogenous bone graft, which is the gold standard for repairing bone defects, in the same experimental model [32,33]. These results demonstrate that chemical modification of gelatin with EGCG has a great potential to increase the bone forming capability of gelatin. However, it is still unknown whether this chemical modification strategy is applicable in the scaffold preparation used in stem-cell-based bone tissue regeneration.

Multipotent progenitor cells, such as adipose-derived stem cells (ADSCs) and dedifferentiated fat (DFAT) cells, are prospective cell sources for various regenerative therapies [34]. These two cell types are both isolated from the same fat tissue, but by different methods [35]. Adipose-derived stem cells are traditionally prepared from stromal vascular fractions (SVFs) via centrifugation of fat tissue; DFAT cells are dedifferentiated from mature adipose cells by the ceiling culture or the preincubation-filter method [35]. The surface antigens and proliferative capacity of DFAT cells are similar to those of mesenchymal stem cells and ADSCs, but are more homogenous [35,36]. For bone formation, the implantation of ADSC has improved the osteoconduction in created (non-congenital) bone defects in rat calvaria [37] and facilitated ectopic bone formation in the mouse subcutaneous region [38]. More recently, Cho et al. [31] reported that the simple coating of EGCG with polycaprolactone slightly potentiated the bone formation of ADSC in critical size defect of calvaria in mice. Dedifferentiated fat cells have augmented bone formation in a created bone defect in alveolar bone [7,39]. These two cell types have been proved to differentiate into osteoblastic cells in vitro by the stimulation of growth factors and small molecules [38,40]. Dedifferentiated fat cells differentiate into osteoblasts much earlier than bone marrow-derived mesenchymal stem cells or ADSC [41,42]. Furthermore, our previous study [40] proved that EGCG can induce the differentiation of human DFAT cells into osteoblastic cells in vitro. Thus, taken together, these findings indicate that gelatin chemically modified with EGCG (vhEGCG-GS) holds the potential to promote bone formation, even in congenital bone defects, when used with ADSC or DFAT cells in vivo. 

Therefore, in the present study, we evaluated whether vhEGCG-GS with/without rat ADSC or DFAT cells separately (hereafter rADSC and rDFAT cells) induces bone formation in a rat congenital cleft-jaw model. The experimental model has been recently developed using the rat mandibular symphysis composed of fibrous tissue and physiological bone gap [43]. To the best of our knowledge, this study is the first to evaluate the osteogenesis of rADSC and rDFAT cells in this new experimental model. Furthermore, to elucidate the mechanism underlying the bone-forming ability of vhEGCG-GS, we compared the water wettability, zeta potential, cell attachment property, and calcium precipitation of the sponges in vitro.

## 2. Results

### 2.1. Work Flow of Cell Implantation

**Figure 1:**
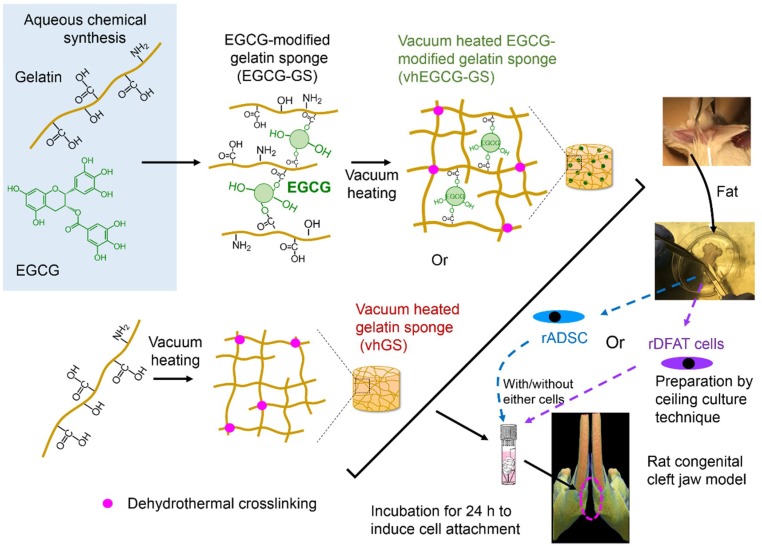
Scheme showing the workflow for the preparation of sponges and cell transplantation. rADSC: rat adipose-derived stem cells; rDFAT cells: rat dedifferentiated fat cells; ECGC: epigallocatechin gallate.

**Figure 2:**
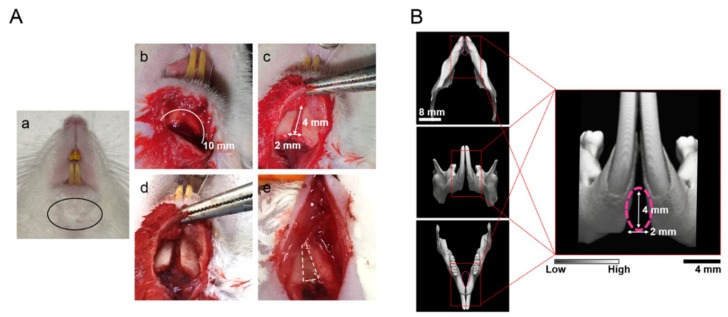
Rat congenital cleft-jaw model. (**A**) The surgical procedure to prepare the congenital cleft-jaw model in the mandibular symphysis. (**A-a**) Black eclipse: incision part. (**A-b**) White line: incision line to disclose the mandibular symphysis. (**A-c**) The fibrous tissue in the mandibular symphysis (4 × 2 mm was removed by the sharp curettes). (**A-d**) Prepared bone defect for the implantation. (**A-e**) Representative image of implanted vhEGCG-GS. (**B**) Pink broken circle: micro-computed tomography (µCT) image of the congenital bone defect in the mandible symphysis.

Figure 1 shows the work flow used for the material preparation and cell implantation in the present study. After the preparation of two sponges, rADSC or rDFAT cells were seeded on both sponges, cultured for 24 h, and transplanted into the cleft of the mandible (Figure 2). The bone formation induced by the single use of sponges (vhEGCG-GS or vhGS) was also evaluated.

### 2.2. Characteristic of Sponges

**Figure 3:**
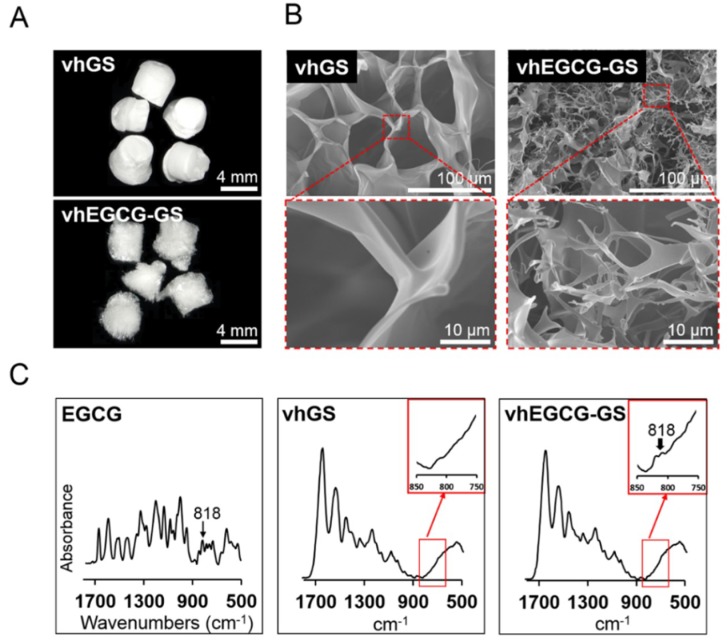
Characterization of the prepared sponges. (**A**) Macroscopic images and (**B**) scanning electron microscopy (SEM) images of the sponges. (**C**) Fourier transform infrared (FTIR) spectra of the sponges and EGCG.

Figure 3A,B shows the macroscopic and scanning electron microscopy (SEM) images of the prepared vhGS and vhEGCG-GS. Both sponges had a spongy structure, although vhEGCG-GS exhibited smaller pores than did vhGS. We identified the existence of EGCG spectra (818 cm^−1^) in the vhEGCG-GS spectra measured using attenuated total reflection Fourier-transform infrared (ATR-FTIR) spectroscopy (Figure 3C) [32]. 

### 2.3. Identification of Adipose-Derived Stem Cells and Dedifferentiated Fat Cells

**Figure 4:**
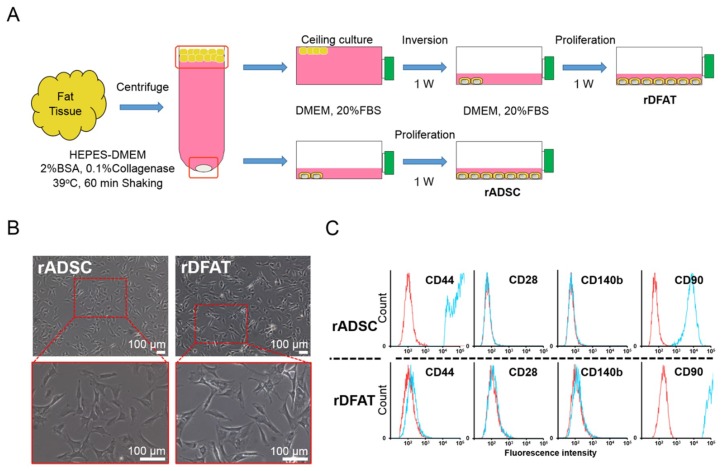
Preparation of the two types of multipotent progenitor cells. (**A**) Scheme of the preparation of the rADSC and rDFAT cells. (**B**) Microscopic view of rADSC and rDFAT cells at first passage. (**C**) Surface antigens on rADSC and rDFAT cells.

The rADSC and rDFAT cells were isolated from fat tissue using centrifugation and ceiling culture technique (Figure 4A). There were negligible differences in cell morphology between the rADSC and rDFAT cells, as observed by phase contrast microscopy (Figure 4B). Meanwhile, there were obvious differences in the surface antigens, as determined by the FACS analysis, especially with respect to the CD90 and CD44 (Figure 4C), suggesting that the two isolated cells have different cell surface characteristics. 

### 2.4. Bone Formation in Rat Congenital Cleft-Jaw Model

**Figure 5:**
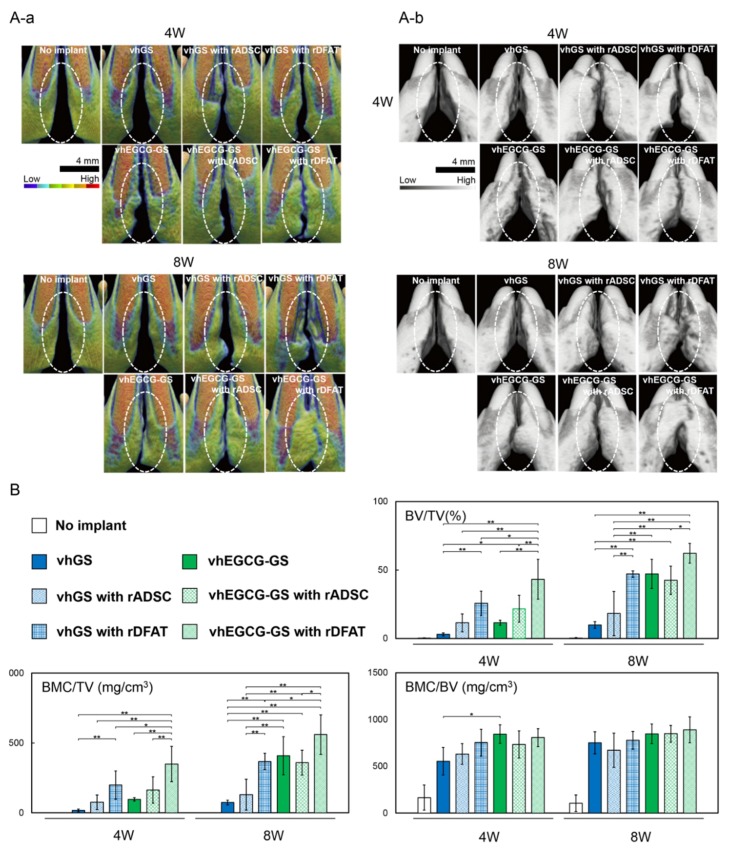
Micro-computed tomography (µCT) analysis of new bone formation in the congenital cleft-jaw model. (**A-a**) Representative bone mineral density image of the defects at 4 and 8 weeks after the surgery; anterior view. (**A-b**) Representative µCT images of the defects at 4 and 8 weeks after the surgery; diagonally upward view. (**B**) Bone morphometric analysis. BV: bone volume; TV: total volume; BMC: bone mineral content. * *p* < 0.05, ** *p* < 0.01 (one-way ANOVA with a Tukey–Kramer test; All statistical significance except for the comparison against no implant was highlighted). The bar graph shows the mean with standard deviation (*n* = 5).

**Figure 6:**
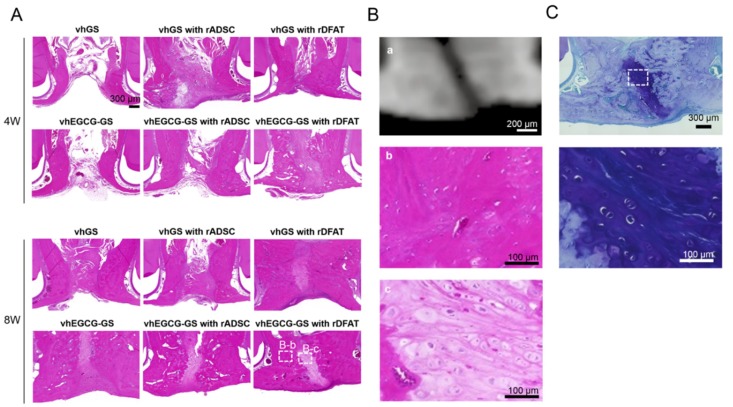
Representative histological and radiological images of the bone defects. (**A**) Low magnification of sections stained with hematoxylin-eosin (H-E). White squares: magnified area used in B-b and c. (**B-a**) Cross-section of µCT images approximately coincided with H-E staining of vhEGCG-GS with rDFAT cells at 8 weeks. (**B-b**,**c**) High-magnification images of H-E staining of vhEGCG-GS with rDFAT cells at 8 weeks. (**C**) Low- and high-magnification images of toluidine blue staining of vhEGCG-GS with rDFAT cells at 8 weeks. White squares: magnified area.

Using the cells isolated by the method described above, we implanted the vhEGCG-GS and vhGS with/without the cells (Figure 1). When implanted with cells, rADSC and rDFAT cells were individually implanted with sponges. The bone morphometric analysis using µCT and histological evaluation (Figure 5 and Figure 6) revealed that there was limited bone formation (bone volume/total volume: BV/TV) in the defect without sponges and with vhGS up to 8 weeks after implantation (Figure 5B). The combination of rADSC or rDFAT cells with sponges potentiated bone formation in the defects from 4 weeks (Figure 5A,B). The vhEGCG-GS with rDFAT cells showed greater ossification (BV/TV) at 8 weeks (Figure 5B). Histological observation confirmed that the increased increment in radiopacity was due to the newly formed bone (Figure 6A,B-b). Interestingly, the use of vhEGCG-GS alone (only sponges) could induce superior ossification at 8 weeks, despite the lack of rADSC and rDFAT cells. A previous study has reported that cartilage could be formed in the rat cleft jaw when implanted with the bone marrow stromal cells with/without β-tricalcium phosphate [43,44]. Concordant with these results, we found that cartilage tissue emerged in the bone defect treated with both rADSC and rDFAT cells (Figure 6B-c,C and Table 1). However, increased cartilage tissue could be mostly observed in the defect treated with vhEGCG-GS (with/without cells) (Table 1).

### 2.5. In Vitro Cell Attachment Assay

**Figure 7:**
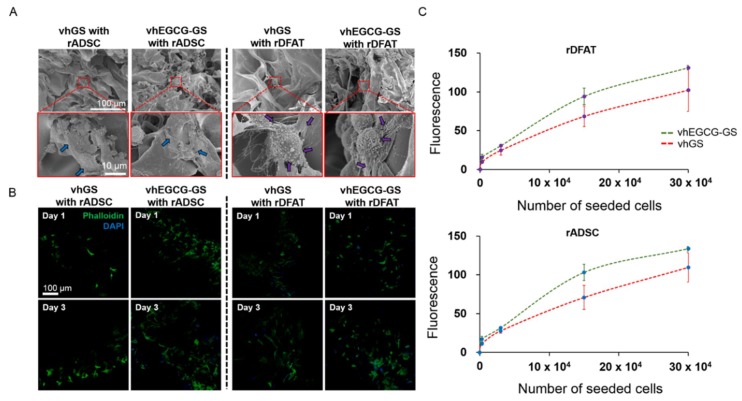
Cell attachment property of the sponges. (**A**) Low- and high-magnification SEM images of the cells cultured on the sponges for 24 h. Blue Arrows: rADSC; purple arrows: rDFAT cells. (**B**) Immunostaining of the cells cultured on the sponges for 1 and 3 days. Green: phalloidin staining; Blue: DAPI staining. (**C**) Semi-quantitative analysis of the cell number on the sponges evaluated with Picogreen assay at day 1. Cells were seeded at various cell densities and cultured for 24 h. *y*-axis: fluorescence, *x*-axis: seeded cell number. The dots show the mean with standard deviation (*n* = 2).

To elucidate the mechanism underlying the induction of superior bone formation by vhEGCG-GS with rDFAT cells than by vhGS with cells, the cell attachment behavior of both cells was evaluated using vhGS and vhEGCG-GS in vitro. rADSCs and rDFAT attached to both sponges and showed a spreading morphology (Figure 7A). There were negligible differences between the morphology of the cells on both sponges. However, rADSC and rDFAT cell attachment was higher on vhEGCG-GS than on vhGS (Figure 7B,C). The results suggest that the vhEGCG-GS could enable efficient cell attachment during transplantation.

### 2.6. Evaluation of Surface Property on Sponges

**Figure 8:**
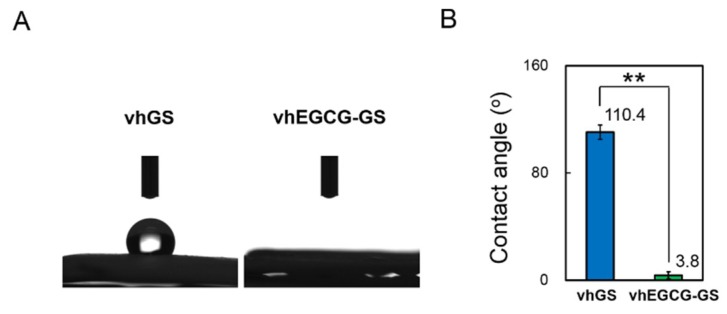
Water wettability of the membrane prepared from vhGS and vhEGCG-GS. (**A**) Macroscopic images. The water droplet was 1 µL. (**B**) Water contact angle of the membrane. Data were obtained at 15 s after the water drop. ** *p* < 0.01 (Student’s t test). The bar graph shows the mean with standard deviation (*n* = 12). Numbers: means of contact angles.

**Figure 9:**
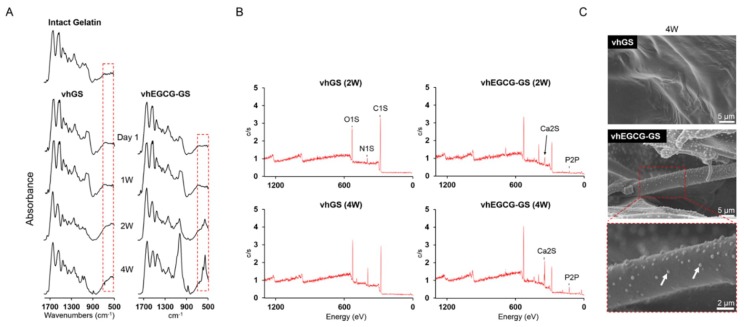
Calcium phosphate precipitation on the sponges immersed in Dulbecco’s modified Eagle’s media for up to 4 weeks. (**A**) FTIR spectra, (**B**) X-ray photoelectron spectra, and (**C**) SEM images of sponges. (**C**) White arrows: precipitated calcium phosphate.

To characterize the mechanism underlying the increased attachment of rADSC and rDFAT cells to vhEGCG-GS compared to vhGS, we investigated the water wettability, zeta potential, and mineralization of both sponges in vitro (Figure 8, Figure 9, Figure S1 and S2).

The vhGS exhibited a hydrophobic surface (110.4°), while vhEGCG-GS exhibited a hydrophilic surface (3.8°) (Figure 8). The zeta potential of vhGS was +0.24 mV, while that of vhEGCG-GS was −0.54 mV. We could not detect any mineralization on both sponges by 1-week immersion in cell culture medium (Figure 9A and Figure S2). After immersion for 2 weeks, the phosphate spectra (558 cm^−1^) started emerging only in the spectra of vhEGCG-GS. Using XPS analysis, we confirmed the calcium and phosphate peaks in the spectra of immersed vhEGCG-GS (Figure 9B). In contrast to the surface of vhGS (no EGCG), SEM analysis revealed small dots on the surface of the vhEGCG-GS (Figure 9C). These results provide evidence that vhEGCG-GS undergoes mineralization in the culture medium with time, compared with vhGS.

## 3. Discussion

Despite the great demand for treating craniofacial bone defects, functional and cost-effective scaffolds capable of inducing ossification by multipotent progenitor cells remain unestablished [8]. The present study demonstrated that vacuum-heated gelatin chemically modified with EGCG (vhEGCG-GS) induced superior bone formation, when used with rDFAT cells or rADSC than did vhGS (without EGCG) with the two types of cells or the sponges alone in a rat congenital cleft-jaw model. The vhEGCG-GS enabled efficient attachment of rDFAT cells and rADSC compared with vhGS. The surface characteristics of vhEGCG-GS were remarkably differed from those of vhGS, with respect to the water wettability, zeta potential, and mineralization. The results strongly suggest that chemical modification of gelatin by EGCG may not only provide pharmacological effects, but also alter the physicochemical properties of the base material (gelatin).

So far, there are a number of reports evaluating the bone-forming ability of biomaterials using rat models, such as bone defects in calvaria [1,29,33], jaw [39], and long bone [45]. Those defects were surgically created in pre-existing bone tissue. These experimental models were undoubtedly beneficial in evaluating the bone-forming ability of novel biomaterials. However, all of these models elucidated osteoconductivity and not ectopic ossification. In order to understand congenital bone defects, such as cleft lip and palate, congenital bone-defect models are essential. The subcutaneous implant model is a promising candidate; however, this model is far from ready for clinical case studies. Recently, Yaguu et al. [43] established the beneficial use of rat mandibular symphysis as a congenital cleft-jaw model. We utilized this model in the present study for evaluating the bone-forming ability of vhEGCG-GS with two types of multipotent progenitor cells (Figure 1 and Figure 2). To the best of our knowledge, there is no other study evaluating the osteogenesis of rADSC and rDFAT cells using this model. Although we could not directly extrapolate our results to the treatment of human cleft jaw, our results indicate that vhEGCG-GS might be a useful scaffold for inducing ossification by both types of multipotent progenitor cells, even in the case of a congenital bone defect.

Non-differentiated multipotent progenitor cells are occasionally used to assess bone regeneration therapy effects [37], although various studies have differentiated cells towards the osteoblastic lineage using osteogenic media in vitro before cell transplantation [6,43]. In the present study, we transplanted the two types of progenitor cells without osteogenic induction in vitro, keeping in mind the issue of the cost of future clinical application. However, both sponges with non-differentiated rADSC and rDFAT cells successfully induced better bone formation than the use of the sponges alone at four weeks (Figure 5). Additionally, the combination of cells with vhEGCG-GS induced significantly greater bone formation than vhGS plus both cell types. A previous study has revealed that the polyphenol EGCG induced the differentiation of mesenchymal stem cells into osteoblastic cells in vitro [46]. We have previously reported that the administration of EGCG induced osteoblast differentiation in human DFAT cells in vitro [40]. Taken together, these results suggest that EGCG-modification in vhEGCG-GS retained the pharmacological property of this polyphenol to induce the differentiation of the implanted rADSC and rDFAT cells into osteoblastic cells in vivo, which was partially associated with a better bone formation in the vhEGCG-GS group.

The vacuum-heating technique was utilized to prepare both vhGS and vhEGCG-GS in this study. Nevertheless, the two types of sponges showed completely opposite water wettability (Figure 8); hydrophobic for vhGS and hydrophilic for vhEGCG-GS. Hydrophilicity of amino acids can be classified based on functional groups according to their polarity and charge characteristics [47]. Carboxyl, amine, and hydroxyl groups, with so-called charged side chains, generally contribute to the hydrophilicity of proteins. It is well known that vacuum-heating treatment accelerates the dehydrothermal cross-linking in proteins using the mentioned functional groups [48], possibly leading to the hydrophobic nature of vhGS. On the other hand, vhEGCG-GS exhibited remarkable hydrophilic property. This might be due to the residual hydroxyl group in EGCG in vhEGCG-GS. In fact, the zeta potential of vhEGCG-GS was negative, in contrast to that of vhGS. Prasersung [49] has reported that the dehydrothermal treatment of gelatin hinders cell culture. Therefore, the alterations of the surface characteristics of the scaffold by chemical modification with EGCG is likely to be partially associated with enhancement in cell adhesion and calcium phosphate precipitation.

As mentioned above, more calcium phosphate precipitated on vhEGCG-GS than on vhGS after two weeks of immersion in the cell culture medium. It is known that the surface coating of proteins with calcium phosphate promotes cell proliferation, differentiation, and bone formation [50]. According to the results of the ATR-FTIR analysis of vhEGCG-GS (see spectra around 500–600 cm^−1^ in FTIR spectra; Figure 9A), there were little spectra of phosphate representing calcium phosphate on the sponges after up to one week of immersion in the culture medium. These results indicate that calcium phosphate precipitation may contribute to the bone formation, but not to early cell adhesion.

Although our results indicate that the vhEGCG-GS has the potential to be used as a scaffold for rADSC or rDFAT cells in bone regeneration therapy, further in-depth evaluations are needed to standardize the clinical use of this scaffold. For example, information on the optimal cell-seeding numbers of the two cell types to be used in combination with vhEGCG-GS is unknown. Further, it is important to investigate whether differentiated or undifferentiated cells are the most suitable type of cells for use with vhEGCG-GS; the non-differentiation protocol is more cost-effective. In addition, the optimal dose of EGCG for use in vhEGCG-GS is still unknown in the context of stem-cell-based therapy, although our previous study has already exhibited that the single use of vhEGCG-GS, containing the same dose of EGCG as used in this study, showed significant bone-forming ability compared with that containing other doses of EGCG in the rat critical-sized bone defect model [32]. A comparative study of vhEGCG-GS and other bone substitute materials might offer valuable findings. Lastly, a dental implant is occasionally used for the replacement of missing teeth in the congenital bone defect [51]. It may be necessary to confirm the functional efficiency of the newly formed bone with respect to the use as a dental implant procedure and others. However, given the shortage of autogenous bone grafts, our results indicate that the chemical modification of EGCG on gelatin can be a promising strategy to produce useful scaffolds for stem-cell-based therapy of incurable craniofacial abnormalities.

## 4. Materials and Methods

### 4.1. Preparation of EGCG-GS and vhEGCG-GS

The EGCG-GS was prepared by the aqueous synthesis method reported previously [29]. In brief, type A gelatin (100 mg; Sigma–Aldrich, St. Louis, MO, USA) was dissolved in warm Milli-Q water (5 mL) at 50 °C. The solution with 27.5 µL N-methylmorpholine (NMM), 0.07 mg EGCG, and 69.2 mg 4-(4,6-Dimethoxy-1,3,5-triazin-2-yl)-4-methyl-morpholinium chloride (DMT-MM) was stirred for 24 h at room temperature in the dark. The EGCG was purchased from Bio Verde Inc. (Kyoto, Japan), DMT-MM from Tokyo Chemical Industry Co., Ltd. (Tokyo, Japan), and NMM from Nacalai Tesque Inc. (Kyoto, Japan). The products were dialyzed with Spectra/Por7 MWCO 1000 (Spectrum Labs, Rancho Dominguez, CA, USA) in Milli-Q water in the dark for purification. To prepare the gelatin solution, the same conditions, but without EGCG, DMT-MM, and NMM were used. After dialysis, the resulting solution was diluted to 10 mL with Milli-Q water and was poured in the mold (5 mm diameter, 3 mm height) in a polytetrafluoroethylene plate, followed by pre-freezing for 24 h at −30 °C and lyophilization with DC800 (Yamato Co., Ltd., Tokyo, Japan) to obtain the EGCG-GS or gelatin sponge (GS). The EGCG-GS and GS were treated by vacuum heating using ETTAS AVO-250NS (AS ONE, Osaka, Japan) at 150 °C for 24 h with a gauge pressure of −0.1 MPa to fabricate vhEGCG-GS and vhGS. All sponges were stored at 4 °C in the dark until use.

### 4.2. Characterization of Sponges

The sponges were visualized macroscopically using the EyeSpecial C-III (Shofu Inc., Kyoto, Japan). To evaluate the porous structure, the sponges were observed using scanning electron microscopy (FE-SEM, S-4800; Hitachi, Tokyo, Japan) with an accelerating voltage of 5.0 kV. The samples were coated with OsO_4_ using the HPC-20 osmium coater (Vacuum Device Co., Ltd., Ibaraki, Japan) before observation. The existence of EGCG in vhEGCG-GS was identified by absorbance spectra using ATR-FTIR spectroscopy (Perkin Elmer Spectrum One, PerkinElmer, Inc, Waltham, MA, USA or IRAffinity-1S, Shimadzu, Kyoto, Japan). The sponges were analyzed over a range of 1800 to 500 cm^−1^ with 2 cm^−1^ resolution. The number of scans was 10. Data pre-processing algorithms were used to set the baseline and remove noise from the spectra by smoothing. To assess the zeta potential of vhEGCG-GS and vhGS, membranes were prepared from both sponges using a roller (Shofu Inc.) and evaluated with a zeta-potential and particle size analyzer (ELSZ-2000ZS, Otsuka Electronics Co., Ltd., Hirakata, Japan) according to the manufacturer’s instructions. Monitor particle and 10 mM sodium chloride solution were used for this assay. The membrane-shaped vhEGCG-GS and vhGS were used to analyze water wettability using the contact angle meter (LSE-ME2, Nick Corporation, Kawaguchi, Japan). One microliter of Milli-Q water was dropped onto the membrane, followed by measurements at 15 s.

### 4.3. Cell Preparation of Adipose-Derived Stem Cells and Dedifferentiated Fat Cells

All animal experiments were approved by and strictly conformed to the guidelines of the Local Ethics Committee of Osaka Dental University (Approval No. 17-03003). The rADSC and rDFAT cells were isolated from subcutaneous fat tissue harvested from the inguinal part of Fisher F344 rats (male, 8 weeks old). In brief, minced fat tissues were digested with 4-(2-hydroxyethyl)-1-piperazineethanesulfonic acid (HEPES)-Dulbecco’s modified essential medium (DMEM) containing 2% bovine serum albumin and 0.1% collagenase and then filtered through a nylon mesh to remove undesirable components, such as vascular cells and connective tissues. The filtered solution was centrifuged at 100 × *g* to obtain SVF and mature adipocytes. To prepare rADSC cells, SVF were seeded onto the culture flask and cultured for further experiments. As for the DFAT cells, the ceiling culture method was used for preparing the rDFAT cells. Mature adipocytes containing lipid droplets were seeded onto a culture flask with the adhesive surface facing upward. The floating adipocytes adhered to the inner ceiling of the flask. One week later, fibroblastic cells designated as DFAT cells emerged. The flask was overturned to remove the mature adipocytes. Both cells were maintained in DMEM supplemented with 10% heat inactivated fetal bovine serum (FBS) and 1% antibiotics in a 5% CO_2_ incubator at 37 °C. Cells at first passage were used for all the following experiments. The surface antigens of cells were analyzed with FACSVerse (BD Biosciences, Franklin Lakes, NJ, USA). Cells (2 × 10^5^) were obtained after treatment with 0.25% trypsin (Thermo Fisher Scientific Inc. Waltham, MA, USA). Staining of cell surface antigens was carried out in phosphate buffered saline (PBS) with 2% newborn calf serum (Bovogen Biologicals, Keilor East, VIC, Australia). The cell suspension was incubated with specific antibodies for 30 min at 4 °C. We evaluated the following surface antigens with antibodies: CD44 (Cat#12-0444-80, Thermo Fisher Scientific Inc.), CD28 (Cat#102105, BioLegend, San Diego, CA, USA), CD140b (Cat#323605, BioLegend), and CD90 (Cat#202526, BioLegend).

### 4.4. Cell-Seeding and Cell Attachment Assay

The cells (30 × 10^4^ cells) were seeded onto sterilized 10 columnar sponges (ϕ5 × 3 mm for each sponge) in cryotubes containing DMEM without FBS. To allow the cells to adhere firmly, the tubes were rotated for 1 h and incubated for 24 h. The sponges with cells were used for further animal experiments. For in vitro analysis, 3 × 10^4^ cells were seeded onto 10 sterilized sponges in cryotubes containing DMEM without FBS to distinguish each cell easily. For the SEM observation, cells were washed twice with PBS and fixed using 2% glutaraldehyde in 0.1 M PBS for 24 h at room temperature. The samples were then sequentially dehydrated with various concentrations of ethyl alcohol and coated with OsO_4_ using the HPC-20 osmium coater (Vacuum Device Co., Ltd.). The SEM images were captured with FE-SEM (S-4800, Hitachi) with an accelerating voltage of 5.0 kV. For immunostaining, at the prescribed time points, the cells were fixed with 4% paraformaldehyde phosphate buffer solution. After washing with PBS, the fixed cells were permeabilized with 1% Triton X-100 in PBS. Actin was stained with Acti-sain 488 phalloidin (1:200; Cytoskeleton, Inc., Denver, CO, USA). Nuclear staining and mounting were performed using DAPI-Fluoromount-G (SouthernBiotech, Birmingham, AL, USA). For the quantitative assay, various numbers of cells were seeded onto the sponges. The cell numbers in the sponges were semi-quantitatively analyzed with picogreen dsDNA quantification regent (Thermo Fisher Scientific Inc.) according to the manufacturer’s protocol.

### 4.5. Preparation of Congenital Cleft-Jaw Model and Implantation of Samples

The central portion of the mandible, namely the mandibular symphysis, was composed of fibrous tissue without bone connection [43]. This congenital bone defect of Fisher F344 rats (male, 8 weeks old) was used to evaluate the ossification of each sample (Figure 2). The rats were anesthetized pre-operatively with intraperitoneal injection of a mixture of medetomidine hydrochloride (0.15 mg/kg; Domitor; Zenoaq, Fukushima, Japan), midazolam (2 mg/kg; Midazolam Sandoz, Sandoz K.K., Yamagata, Japan), and butorphanol tartrate (2.5 mg/kg; Vetorphale, Meiji Sika Parma Co., Ltd., Tokyo, Japan). The surgery was performed as reported previously [43]. In brief, an incision was made in the skin at the inferior margin of the mandible. The soft tissue and mandibular symphysis were curetted to create a space (2 mm wide, 4 mm high, and 1 mm deep). Ten columnar sponges with/without cells, after 24 h of cell culture as mentioned above, were implanted into each defect; the skin was laid over the defect and sutured firmly. The defect without implantation was used as the negative control. At 4 and 8 weeks after implantation, the treated mandibles were harvested to verify the bone-forming ability of each sample. Five rats were used for each group: 1, No implant; 2, vhGS only; 3, vhGS with rADSC; 4, vhGS with rDFAT cells; 5, vhEGCG-GS only; 6, vhEGCG-GS with rADSC; and 7, vhEGCG-GS with rDFAT cells. In total, 70 rats were used for the experiments (5 rats × 7 groups including the negative control for 4 and 8 weeks).

### 4.6. Micro-Computed Tomographical and Histological Analysis

To analyze the radiopacity and morphology of the newly formed bone in the defects, the treated mandibles were assessed by micro-computed tomography (µCT) scanning (SMX-130CT; Shimadzu) at 71 μA of 75 kV radiation. Images were saved at a resolution of 512 × 512 pixels. TRI/3D-Bon (Ratoc System Engineering, Tokyo, Japan) was used to reconstruct the anterior and the diagonally upward views of the mandible. Bone mineral density representing calcified bone tissue was assessed using cylindrical phantoms containing hydroxyapatite (hydroxyapatite content: 200–1550 mg/cm^3^). The BV/TV, bone mineral content (BMC)/TV, and BMC/BV were calculated to assess the mineralized tissue volume, weight, and density in the defects. For the histological evaluation, each calvaria was fixed with 4% paraformaldehyde phosphate buffer solution, decalcified with formic acid, dehydrated, and embedded in paraffin. Thin sections (5 μm in thickness) were prepared and stained with hematoxylin–eosin and toluidine blue. All images were captured using the BZ-9000 digital microscope (Keyence Co., Osaka, Japan).

### 4.7. Calcium Phosphate Precipitation

Calcium phosphate coating is known to facilitate the bone-forming ability of biomaterials [52]. Both sponges were incubated in DMEM for 1 day and 1–4 weeks in cryotubes (500 µL per tube) to analyze the calcium phosphate precipitation. At prescribed time points, the treated sponges were evaluated using FE-SEM (S-4800; Hitachi), X-ray photoelectron spectroscopy (XPS; PHI X-tool, ULVAC-PHI, Kanagawa, Japan), and ATR-FTIR spectroscopy (IRAffinity-1S, Shimadzu).

### 4.8. Statistical Analysis

Statistical analyses were performed using Microsoft Excel software (Microsoft Co., Redmond, WA, USA) with the add-in software Statcel 4 (OMS, Saitama, Japan). All numerical data are presented as the mean ± standard deviation. Statistical significance was calculated using the Student’s *t*-test or a one-way analysis of variance, followed by the Tukey–Kramer test. Differences at *p* < 0.05 were considered significant.

## 5. Conclusions

Cost-effective plant polyphenols are widely recognized as promising health-promoting agents owing to their beneficial pharmacological effects. Here, we provide evidence that gelatin chemically modified with EGCG (vhEGCG-GS) can be used as a scaffold that enables rADSC and rDFAT cells to induce ossification in the congenital cleft-jaw model. The vhEGCG-GS exhibited superior bone-forming capability than did vhGS (no EGCG), when used with the two types of progenitor cells. In vitro experiments revealed that the EGCG-modification of gelatin altered the water wettability and zeta potential; the modification augmented cell adhesion; and the precipitation of calcium phosphate. These results indicate that cost-effective EGCG is not only usable as a pharmacological agent for tissue engineering, but is also a promising tool to modify gelatin. This strategy will provide insights into the chemical modification of other polymers used in stem-cell-based regeneration therapy.

## Figures and Tables

**Table 1:** Summary of cartilage formation.

		No EGCG			EGCG-Modification		
	No Implant	vhGS	vhGS with rADSC	vhGS with rDFAT Cells	vhEGCG-GS	vhEGCG-GS with rADSC	vhEGCG-GS with rDFAT Cells
4 weeks	0/5	0/5	1/5	1/5	0/5	0/5	0/5
8 weeks	0/5	0/5	0/5	2/5	3/5	1/5	3/5

When cartilage formation was observed in the section, the number was counted relative to the total number of rats. EGCG: epigallocatechin-gallate; vhGS: vacuum-heated gelatin sponge; vhEGCG-GS: vacuum-heated epigallocatechin-gallate-modified gelatin sponge; rADSC: rat adipose-derived stem cells; rDFAT cells: rat dedifferentiated fat cells.

## Supplementary Materials

Supplementary materials can be found at http://www.mdpi.com/1422-0067/19/12/3803/s1.

## Abbreviations

EGCG	Epigallocatechin gallate
GS	Gelatin sponge
vh	Vacuum heated
EGCG-GS	Epigallocatechin gallate-modified gelatin sponge
vhEGCG-GS	Vacuum heated EGCG-GS
DFAT cell	Dedifferentiated fat cell
ADSC	Adipose-derived stem cell
SEM	Scanning election microscopy
XPS	X-ray photoelectron spectroscopy
FTIR	Fourier transform infrared
BV	Bone volume
TV	Total volume
BMC	Bone mineral content
DAPI	4′,6-diamidino-2-phenylindole
H-E	Hematoxylin and eosin
DMEM	Dulbecco’s modified Eagle’s media
