# Supplementary material for: Osteogenesis of Multipotent Progenitor Cells using the Epigallocatechin Gallate-Modified Gelatin Sponge Scaffold in the Rat Congenital Cleft-Jaw Model
Source: Int J Mol Sci. 2018 Nov 29;19(12):3803. doi: 10.3390/ijms19123803 (PMC6320852; doi:10.3390/ijms19123803)
Supplement: Supplementary file 1 [file ijms-19-03803-s001.pdf]

Supplementary Materials: Osteogenesis of Multipotent Progenitor Cells using the Epigallocatechin Gallate-Modified Gelatin Sponge Scaffold in the Rat Congenital Cleft-Jaw Model.

Satoshi Sasayama, Tomoya Hara, Tomonari Tanaka, Yoshitomo Honda, and Shunsuke Baba.

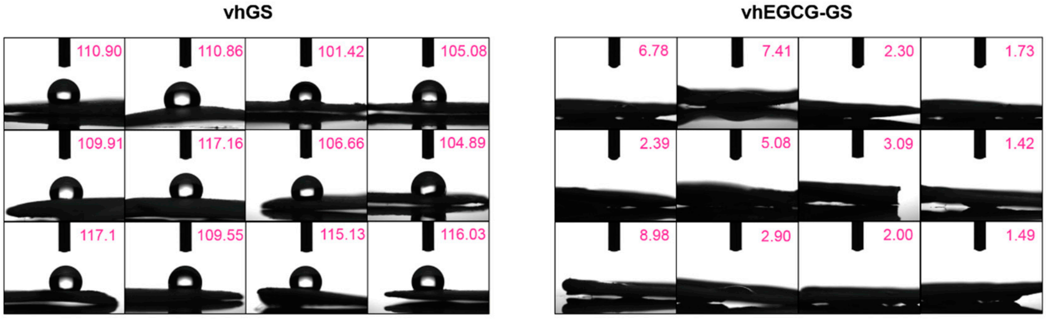

Figure S1. Water wettability of the membrane prepared from vhEGCG-GS and vhGS. All 24 samples are shown.

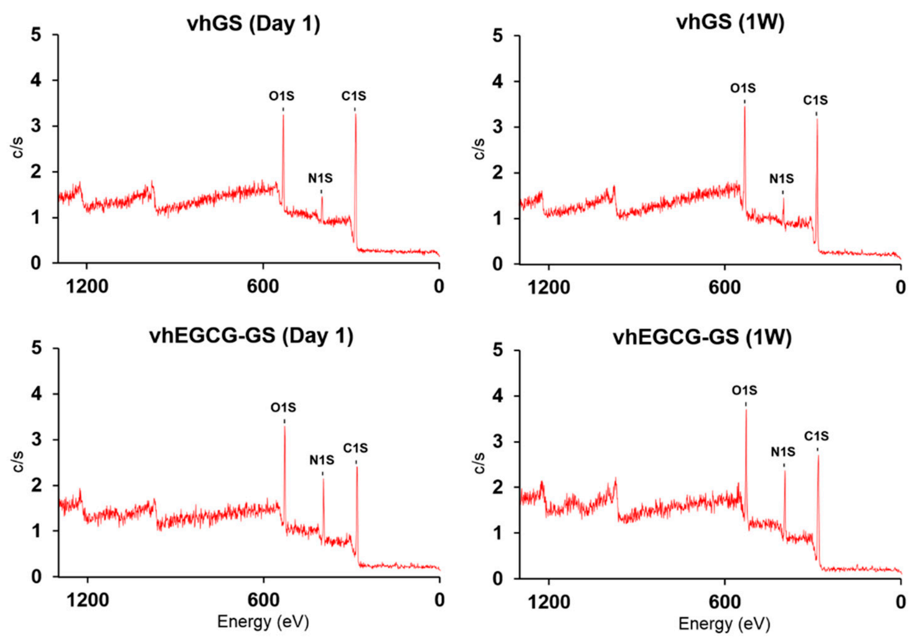

Figure S2. X-ray photoelectron spectroscopy spectra of vhEGCG-GS and vhGS immersed in the Dulbecco's modified essential medium for 1 day or 1 week.
